# Prion Replication in the Mammalian Cytosol: Functional Regions within a Prion Domain Driving Induction, Propagation, and Inheritance

**DOI:** 10.1128/MCB.00111-18

**Published:** 2018-07-16

**Authors:** Yvonne Duernberger, Shu Liu, Katrin Riemschoss, Lydia Paulsen, Romina Bester, Peer-Hendrik Kuhn, Manuel Schölling, Stefan F. Lichtenthaler, Ina Vorberg

**Affiliations:** a German Center for Neurodegenerative Diseases (DZNE), Bonn, Germany; b Institute of Virology, Technical University of Munich, Munich, Germany; c German Center for Neurodegenerative Diseases (DZNE), Munich, Germany; d Institute of Pathology, Technical University of Munich, Munich, Germany; e Institute for Advanced Study, Technical University of Munich, Garching, Germany; f Department of Neuroproteomics, Klinikum rechts der Isar, Technical University of Munich, Munich, Germany; g Munich Cluster for Systems Neurology (SyNergy), Technical University of Munich, Munich, Germany; h Department of Neurology, Rheinische Friedrich Wilhelms University of Bonn, Bonn, Germany

**Keywords:** amyloid, neurodegeneration, prion-like, prions, protein misfolding

## Abstract

Prions of lower eukaryotes are transmissible protein particles that propagate by converting homotypic soluble proteins into growing protein assemblies. Prion activity is conferred by so-called prion domains, regions of low complexity that are often enriched in glutamines and asparagines (Q/N). The compositional similarity of fungal prion domains with intrinsically disordered domains found in many mammalian proteins raises the question of whether similar sequence elements can drive prion-like phenomena in mammals. Here, we define sequence features of the prototype Saccharomyces cerevisiae Sup35 prion domain that govern prion activities in mammalian cells by testing the ability of deletion mutants to assemble into self-perpetuating particles. Interestingly, the amino-terminal Q/N-rich tract crucially important for prion induction in yeast was dispensable for the prion life cycle in mammalian cells. Spontaneous and template-assisted prion induction, growth, and maintenance were preferentially driven by the carboxy-terminal region of the prion domain that contains a putative soft amyloid stretch recently proposed to act as a nucleation site for prion assembly. Our data demonstrate that preferred prion nucleation domains can differ between lower and higher eukaryotes, resulting in the formation of prions with strikingly different amyloid cores.

## Abstract (precis)

Prions of lower eukaryotes are transmissible protein particles that propagate by converting homotypic soluble proteins into growing protein assemblies. Prion activity is conferred by so-called prion domains, regions of low complexity that are often enriched in glutamines and asparagines (Q/N).

## INTRODUCTION

Prions are infectious protein aggregates that propagate by autocatalytically converting normal cellular prion proteins into ordered protein assemblies with cross-β structure, so-called amyloids. The only bona fide prions identified so far in mammals cause fatal transmissible spongiform encephalopathies (TSE) (1). The cellular isoform of the mammalian prion is PrP^C^, a protein tethered to the cell membrane by a glycosylphosphatidylinositol anchor. Its conformational conversion generates infectious entities capable of transmitting between cells, within species, and sometimes even between species (2). Amyloid formation is also a common feature of neurodegenerative diseases of noninfectious origin (3). While epidemiological evidence is lacking that classical neurodegenerative diseases are transmissible, the mechanism of intra- and intercellular molecular templating is shared by many protein aggregates (4).

In analogy to mammalian prions, several proteins of Saccharomyces cerevisiae can adopt self-replicating prion conformations that induce heritable phenotypic traits (5, 6). These fungal protein aggregates are also termed prions and replicate by a mechanism of seeded polymerization in which a seed of a misfolded protein templates the conversion of the soluble protein into a self-perpetuating amyloid state. Fragmentation of existing prion fibrils by the yeast chaperone machinery then leads to the formation of new seeds and exponential multiplication of heritable entities (7, 8). The prion domains (PrDs) of most identified yeast prions are inherently disordered and enriched for asparagine (N) and/or glutamine (Q) residues, with charged and hydrophobic residues being underrepresented (9–13). The compositional similarity of PrDs of known S. cerevisiae prions encouraged the development of computational tools that successfully identified similar domains in several yeast proteins with unknown prion propensity (10, 13–16). Surprisingly, scoring of entire proteomes with prion algorithms predicts that at least 1% of mammalian proteins contain similar prion-like domains (PrLDs) (17, 18). Several mammalian proteins with predicted PrLDs drive liquid-liquid phase transitions for the transient formation of membrane-less ribonucleoprotein complexes. Mutations in the respective domains of disease-associated human proteins have been linked to muscular and neurodegenerative pathomechanisms (19). In light of the many Q/N-rich proteins in higher eukaryotes, it is possible that prion-like self-replication underlies other unresolved epigenetic phenomena and diseases of unknown etiology. So far, however, evidence for bona fide prions derived from human proteins with predicted PrLDs is lacking. Indeed, a recent study demonstrates limitations of prion algorithms to accurately predict the prion propensity of prion-like proteins in higher organisms, likely because host-dependent intracellular factors influence aggregation or prion behavior of a given protein (20).

On a cellular level, prion characteristics include rare spontaneous or template-assisted conversion of the protein into its prion conformation, multiplication of seeds, and natural infection of bystander cells (21, 22). Proof of principle that a prototype yeast prion domain can behave as an infectious entity in mammalian cells comes from studies on the aggregation behavior of the best-studied S. cerevisiae prion, Sup35, in mouse cells (23–25). In yeast, Sup35 serves as a translation termination factor that rarely switches into an inactive prion conformation (26, 27). Its PrD N domain drives prion propagation and assembles into fibrils with cross-β structure *in vitro* (28–31). While the amino acid composition of the N domain is a major determinant of its activity, distinct subdomains within the N domain exert specific but somewhat overlapping functions in prion biogenesis in S. cerevisiae (12, 32, 33). The highly charged middle (M) domain (amino acids [aa] 124 to 250) stabilizes the prion conformer during yeast mitosis and meiosis (34) and increases solubility of the protein in its non-prion state (35). The carboxy-terminal C domain (residues 251 to 685) mediates translation termination function but is otherwise dispensable for prion formation (35, 36). Sup35 NM does not share sequence homology with mammalian proteins and is thus ideally suited to study prion behavior in the absence of a potential loss-of-function phenotype. In analogy to prion induction in S. cerevisiae (37, 38), cytosolically expressed NM adopts a prion state in mammalian cells upon exposure to exogenous *in vitro*-fibrillized recombinant NM (23). Induced NM aggregates behave as infectious entities that faithfully transmit vertically and horizontally (24, 39). Here, we set out to define the subdomains in the Sup35 PrD that mediate the sequential steps of prion formation in mouse neuroblastoma cells. We demonstrate that the differential steps of prion biogenesis are preferentially driven by a carboxy-terminal region of the N domain. Importantly, this domain contains a soft amyloid core proposed to mediate first intermolecular contacts during prion assembly (40). Initiation of prion assembly by the carboxy-terminal region leads to the formation of NM prions that differ strikingly from wild-type prion variants propagating in S. cerevisiae.

## RESULTS

### The Sup35 N domain drives aggregation in mammalian cells.

**FIG 1:**
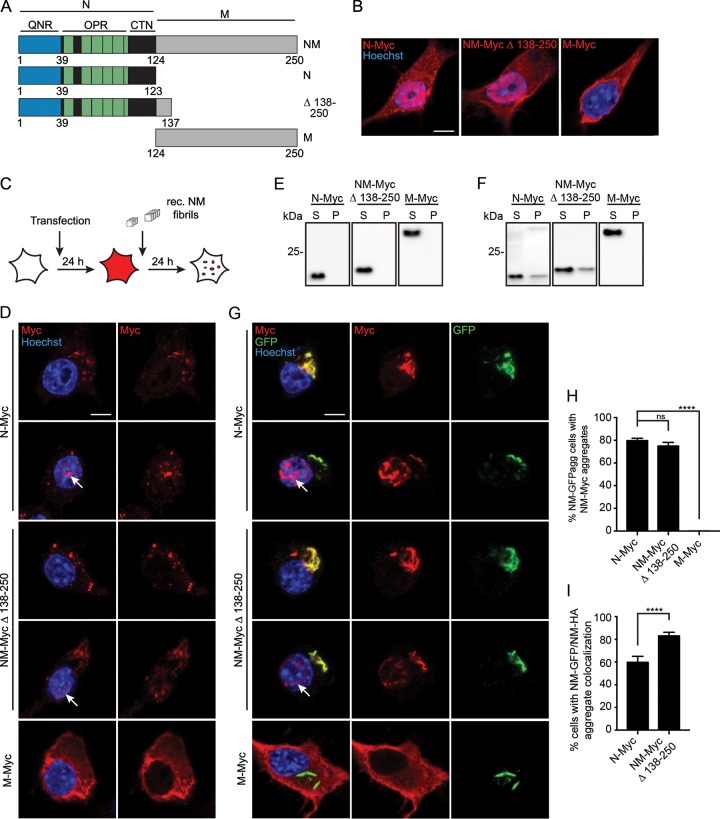
N domain is sufficient for aggregate formation. (A) Schematic diagram of NM constructs. (B) N2a cells 48 h posttransfection of NM constructs. Ectopically expressed proteins were detected using pAb anti-Myc tag (red). Nuclei were stained with Hoechst (blue). Scale bar, 5 μm. (C) Experimental setup to study NM aggregate induction by untagged recombinant (rec.) NM fibrils. Wild-type N2a cells were transiently transfected with constructs N-Myc, NM-Myc Δ 138-250, and M-Myc. Twenty-four hours later, cells were exposed to untagged recombinant NM fibrils. (D) N2a cells transiently transfected with NM mutants and 24 h later exposed to 10 μM (monomer concentration) *in vitro*-fibrillized untagged NM. Ectopically expressed proteins were detected using pAb anti-Myc tag (red). Nuclei were stained with Hoechst (blue). (E) Sedimentation assay of cell lysates from N2a cells 24 h posttransfection with constructs. S, supernatant; P, pellet. Ectopically expressed protein was detected using MAb anti-Myc. (F) Sedimentation assay following exposure to 10 μM fibrillized untagged recombinant NM (monomer concentration). Cells were harvested 48 h posttransfection. The M domain alone exhibits an abnormal migration behavior, potentially due to net charge and/or charge distribution. (G) N2a cell clone 2CG11 stably producing NM-GFP aggregates was transiently transfected with NM mutants for 48 h. NM encoded by plasmids was detected using pAb anti-Myc tag (red). GFP is shown in green. Nuclei were stained with Hoechst (blue). Arrows indicate nuclear NM. Scale bar, 5 μm. (H) N2a NM-GFP^agg^ cell clone 2CG11 was transiently transfected with constructs and fixed 48 h posttransfection. Shown is the percentage of NM-GFP^agg^ cells with Myc-tagged aggregates. Bars represent mean values ± SEM (three independent transfection experiments; *n* = 3). At least 300 cells were analyzed per cell population. The Cochran-Mantel-Haenszel test was used (****, *P* ≤ 0.0001; ns, not significant). (I) Percentage of cells that contain Myc-tagged and GFP-tagged NM aggregates (shown in panel H) that show colocalization of NM-Myc and NM-GFP aggregates. Bars represent mean values ± SEM (*n* = 3). Statistical analysis was done using the Cochran-Mantel-Haenszel test (****, *P* ≤ 0.0001).

We have recently shown that the Sup35 NM domain exhibits prion properties in mammalian cells (23, 24). Here, we use the term “prion” to describe the self-templating and intercellular transmission properties of Sup35 NM in our cellular model in analogy to the well-established fungal prions (41). Myc epitope-tagged N or M domains and a mutant coding for NM Δ 138-250, shown to maintain the prion phenotype in S. cerevisiae (Fig. 1A) (42), were tested for their ability to aggregate upon induction by untagged recombinant NM fibrils or by endogenous green fluorescent protein (GFP)-tagged NM prions. Transiently transfected N2a cells showed diffuse expression of the Myc-tagged mutants in the cytoplasm (Fig. 1B). Proteins lacking parts of the M domain also localized to the nucleus. The reason for the presence of N derivatives in the nucleus is unknown, as the protein lacks a predicted classical or proline-tryosine nuclear localization signal (43). Exposure of cells to *in vitro*-fibrillized untagged recombinant NM revealed that the N domain was crucial for aggregate induction by exogenous NM seeds (Fig. 1C and D). Myc-tagged mutants with deletions in the M domain aggregated in cytoplasm and nuclei. In contrast, M alone was incapable of forming aggregates. A sedimentation assay of cell lysates from transfected cells confirmed that before (Fig. 1E) or after (Fig. 1F) exposure to preformed untagged recombinant NM fibrils, M-Myc expressed in N2a cells remained soluble. When N2a cell clone 2CG11 stably producing NM-GFP prions (24) was transfected with plasmids coding for the Myc epitope-tagged NM mutants (Fig. 1G), aggregation was only observed when the N domain was present, suggesting that, in analogy to the S. cerevisiae system, NM aggregation in mammalian cells is mediated by the same domain (Fig. 1H). Significantly more cells with double-stained NM-GFP aggregates were found for NM-Myc Δ 138-250 than for N-Myc (Fig. 1I). Thus, while the M domain alone is incapable of forming aggregates, a stretch of amino-terminal amino acid residues in M engages in the binding to preexisting NM-GFP prions (Fig. 1I). This is consistent with the finding that residues 124 to 137 are important for variant-specific maintenance of Sup35-derived prions in yeast (42).

### Deletion of the Q/N-rich region increases the rate of spontaneous aggregation.

**FIG 2:**
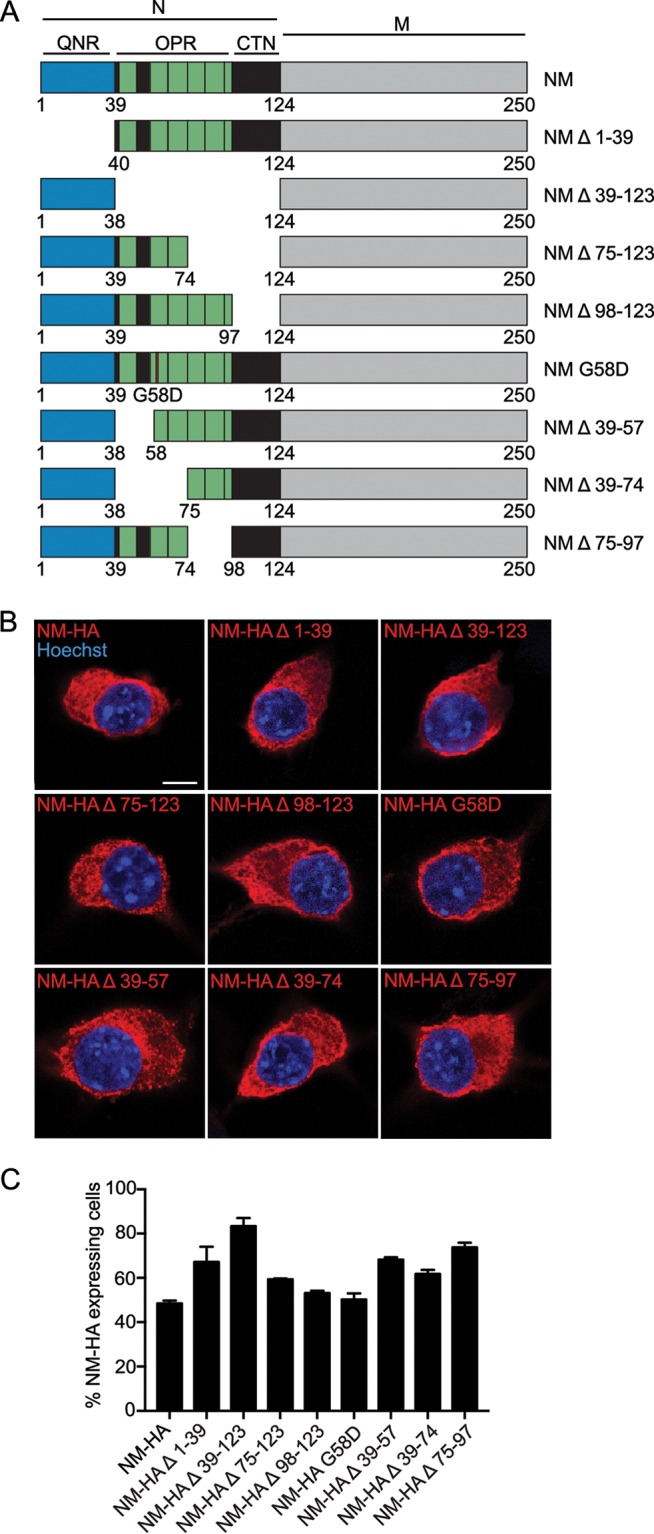
N2a cells stably express NM wild type and NM mutants. (A) Eight NM constructs were generated based on NM mutants already analyzed in S. cerevisiae for their prion propensity (32). QNR, residues 1 to 39; OPR, residues 40 to 97, containing repeats R1 to R5; R1, residues 41 to 49; R2, residues 56 to 64; R3, residues 65 to 74; R4, residues 75 to 83; R5, residues 84 to 93; R5.5, residues 94 to 97; CTN, residues 98 to 123. Note that the terms Q/N-rich region and Q/N-rich tract are used in the field to refer to this region of N (32, 50, 84). The carboxy-terminal region of N also is enriched for Q/N, albeit to a lesser degree. All constructs were tagged with an HA antibody epitope tag at the carboxy termini. (B) Bulk populations of N2a cells stably expressing wild-type and mutant NM-HA. NM was detected using MAb anti-HA (red). Nuclei were stained with Hoechst (blue). Scale bar, 5 μm. (C) Percentage of cells in the bulk population (passage one postthawing) expressing wild-type or mutant NM. Bars represent mean values ± SEM (*n* = 3). At least 14,000 cells were analyzed per cell population.

**FIG 3:**
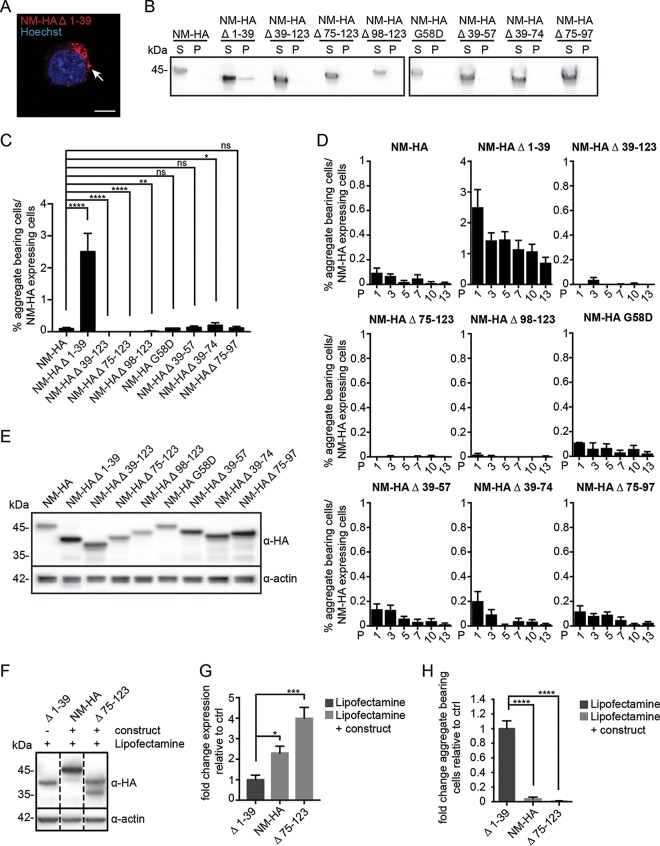
Spontaneous aggregation propensities of full-length and mutant NM in the mammalian cytosol. (A) N2a NM-HA Δ 1-39 cell with spontaneously formed NM-HA aggregates. NM was detected using MAb anti-HA. The nucleus was visualized with Hoechst (blue). The arrow indicates NM-HA aggregates. Scale bar, 5 μm. (B) Sedimentation assay of cell lysates from N2a bulk populations expressing wild-type or mutant NM-HA. NM was detected using MAb anti-HA. S, supernatant; P, pellet. (C) Percentage of cells in the bulk population (one passage postthawing) that express the transgene and also spontaneously form NM aggregates. Bars represent mean values ± SEM from three independent experiments (*n* = 3), with at least 14,000 cells imaged per cell population. Statistical analysis was done using the Cochran-Mantel-Haenszel test (*, *P* ≤ 0.05; **, *P* ≤ 0.01; ****, *P* ≤ 0.0001; ns, not significant). (D) Percentage of N2a NM-HA cells that express wild-type or mutant NM-HA and that spontaneously form NM aggregates over continuous culture (passage 1 to 13 postthawing). Bars represent mean values ± SEM (*n* = 3). At least 9,800 cells were imaged per cell population per passage. Please note variations in scaling. P, passage. (E) Expression of wild-type and mutant NM-HA in stable cell populations. NM was detected by Western blotting using MAb anti-HA. Actin served as a loading control. These data can be compared to the graph shown in Fig. 2C. (F) Stable cell populations expressing full-length NM-HA or NM-HA Δ 75-123 were transiently transfected with constructs coding for the homotypic proteins to increase the expression level of the transgene. N2a NM-HA Δ 1-39 cells were mock transfected. Overexpressed NM-HA Δ 75-123 was partially amino-terminally deleted, as has been observed previously for NM (85). NM was detected using MAb anti-HA. Actin served as a loading control. Additional lanes were excised for presentation purposes. (G) Quantitative analysis of expression shown in panel F (three independent transfections, *n* = 3). Shown is the fold difference in expression level compared to that of mutant NM-HA Δ 1-39. For NM-HA Δ 75-123, only the signal of the upper band was used for quantification. Bars represent mean values ± SEM. Statistical analysis was performed using one-way analysis of variance (*, *P* ≤ 0.05; ***, *P* ≤ 0.001). (H) Spontaneous aggregation of NM-HA and NM-HA mutants in cells shown in panels F and G. Aggregation was assessed 48 h posttransfection. At least 6,000 cells were analyzed per cell population. Bars represent mean values ± SEM. Statistical analysis was done using the Cochran-Mantel-Haenszel test (****, *P* ≤ 0.0001).

We constructed a set of truncated NM mutants previously tested in S. cerevisiae for prion activities (Fig. 2A) (32). The N domain contains a region particularly enriched for Q/N (generally termed QNR; residues 1 to 39) that is crucially required for aggregate induction, polymerization, and amyloid core formation in yeast (11, 44). The five and a half degenerate octapeptide repeats (OPR) promote inheritance over multiple cell divisions by stabilizing intermolecular interactions (45, 46). Importantly the OPR also act as a recognition site for the chaperone Hsp104 that mediates fibril fragmentation in yeast (32, 47, 48). A single-amino-acid substitution (G58D) in the OPR has been shown to modulate Sup35 prion heritability by affecting either fragmentation or bidirectional sorting to progeny (49–52). Aggregate persistence in mitotically active cells is taken as a proxy for effective seed multiplication (22). To restrict expression of the NM derivatives to the cytosol, all deletion mutants contained the M domain. In N2a cell populations stably transduced with virus coding for hemagglutinin (HA) epitope-tagged proteins, NM derivatives generally remained soluble (Fig. 2B). Automated confocal microscopy analysis demonstrated stable transgene expression in at least 38% of the cells over a period of 13 cell culture passages (Fig. 2C and data not shown). In rare cases, we noticed cells expressing NM-HA Δ 1-39 that contained visible NM aggregates (Fig. 3A). Sedimentation assays confirmed that NM-HA proteins almost exclusively resided in a soluble state, with the exception of NM-HA Δ 1-39, where a fraction of the protein became insoluble (Fig. 3B). This is in line with our quantitative confocal microscopy analyses of transduced cell populations, demonstrating that up to 2.5% of cells expressing NM-HA Δ 1-39 exhibited NM aggregates, while spontaneous aggregates were found in less than 0.3% of cell populations expressing full-length NM-HA or other mutants (Fig. 3C). The highest numbers of NM-expressing cells with aggregates were also found in N2a NM-HA Δ 1-39 upon continuous passage (Fig. 3D). Lowest spontaneous aggregation was observed when the last 2.5 repeats and/or the carboxy-terminal N region (CTN) was deleted (Δ 39-123, Δ 75-123, and Δ 98-123). When cells were grown for several days, we noticed clusters of N2a cells with NM-HA Δ 1-39^agg^ but not Δ 75-123^agg^, suggesting that spontaneously forming aggregates lacking the QNR were self-sustained (data not shown).

Prion induction in yeast increases with NM expression levels (31). To test if increased expression resulted in higher aggregate induction efficiencies, cell populations stably expressing full-length NM-HA or NM-HA deletion mutant Δ 75-123 (Fig. 3E) were transiently transfected with expression plasmids coding for homotypic NM proteins to achieve higher expression levels (Fig. 3F). Western blot analysis revealed approximately 2- to 4-fold higher NM levels in lysates of N2a NM-HA or NM-HA Δ 75-123 cells than in lysates of N2a NM-HA Δ 1-39 cells (Fig. 3G). Still, NM-HA Δ 1-39 spontaneously formed aggregates at significantly higher rates than NM-HA or NM-HA Δ 75-123 (Fig. 3H). Increasing transgene expression 3- to 4-fold by transient transfection of N2a NM-HA, N2a NM-HA Δ 75-123, or N2a NM-HA Δ 98-123 cell populations with corresponding plasmids only insignificantly increased NM puncta formation, resulting in less than 0.25% positive cells (data not shown). Collectively, these findings suggest that mutant NM-HA Δ1-39 has an increased ability to self-aggregate into prions.

### The last 2.5 repeats and the carboxy-terminal N region are essential for prion induction by preexisting seeds.

**FIG 4:**
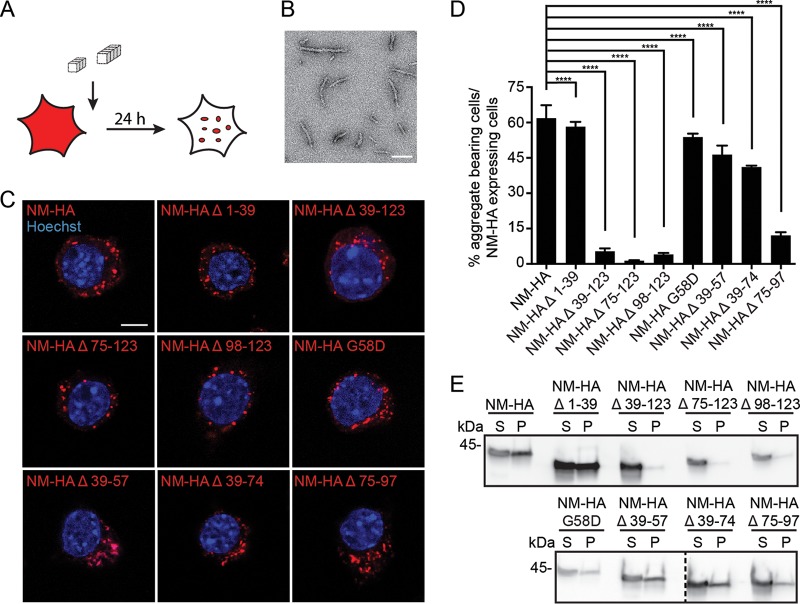
Template-assisted aggregate induction by fibrillized untagged recombinant NM or endogenous NM-GFP prions. (A) Experimental setup to study the propensity of the NM mutants to form aggregates upon exposure to *in vitro*-fibrillized recombinant NM. (B) Electron microscopy image of untagged recombinant NM fibrils. Scale bar, 100 nm. (C) N2a cell populations exposed to 10 μM untagged recombinant NM fibrils (monomer concentration) for 24 h. Ectopically expressed wild-type and mutant NM were detected using MAb anti-HA (red), and nuclei were stained with Hoechst (blue). Scale bar, 5 μm. (D) Percentage of cells harboring NM-HA aggregates upon induction with 10 μM untagged recombinant NM fibrils (monomer concentration). Bars represent mean values ± SEM (three independent induction experiments; *n* = 3). At least 12,500 cells per cell population were imaged. Statistical analysis was done using the Cochran-Mantel-Haenszel test (****, *P* ≤ 0.0001). (E) Sedimentation assay of cell lysates from N2a bulk populations exposed to 10 μM fibrillized untagged recombinant NM (monomer concentration). Cells were harvested 24 h after aggregate induction. Ectopically expressed NM was detected using MAb anti-HA. S, supernatant; P, pellet. Additional lanes were excised for presentation purposes.

To study prion induction by exogenous seeds, N2a cell populations were exposed to untagged recombinant NM fibrils (Fig. 4A and B), and cells were analyzed for NM-HA aggregate induction in passage one (Fig. 4C). Independent of the NM-HA mutant expressed, untagged fibrils seeded protein polymerization in all cell populations (Fig. 4C), albeit at different frequencies (Fig. 4D). Deletion of the QNR (Δ 1-39) or the point mutation G58D only slightly affected numbers of aggregate-bearing cells compared to those of cells that express full-length NM-HA (Fig. 4D). Deletion of the first three repeats (Δ 39-74) had some effect on aggregate induction. In contrast, deletion of the last 2.5 repeats and/or the CTN (Δ 39-123, Δ 75-123, Δ 98-123, and Δ 75-97) drastically impaired aggregation. Sedimentation assays confirmed smaller amounts of insoluble NM-HA when the last 2.5 repeats and/or the CTN were deleted (Fig. 4E).

**FIG 5:**
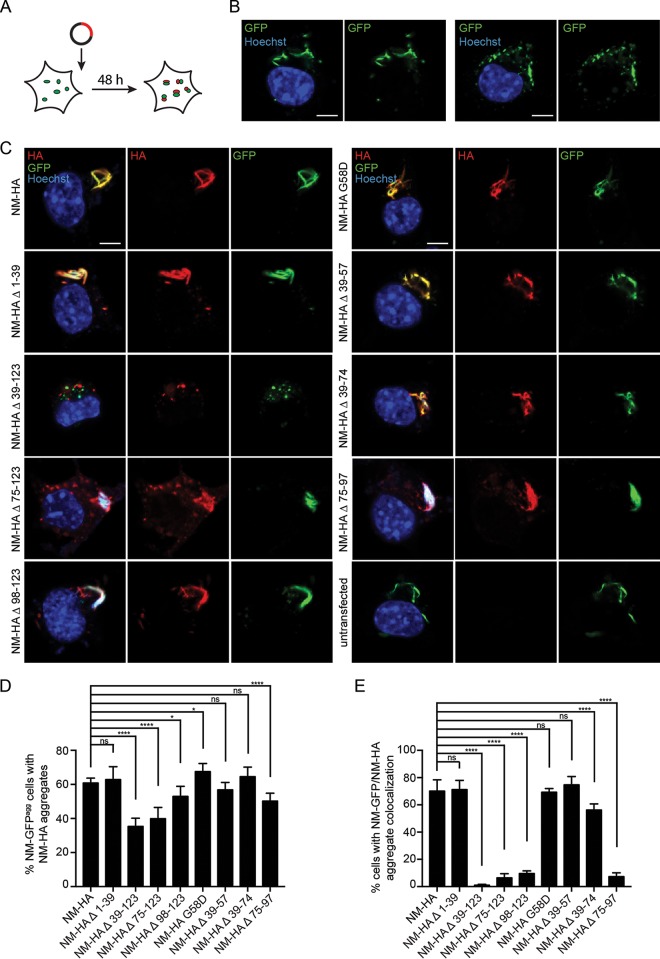
Template-assisted aggregate induction by endogenous NM-GFP prions. (A) Illustration of the experiment. N2a NM-GFP^agg^ cell clone 2CG11 was transiently transfected with empty vector or HA-tagged NM full-length or NM mutant constructs. Cells were fixed 48 h posttransfection. (B) N2a NM-GFP cell clone 2CG11 stably producing NM-GFP aggregates was mock transfected. Nuclei were stained with Hoechst (blue). Note that aggregates can exhibit a fibrillar morphology (left) or exist as puncta (right). Scale bars, 5 μm. (C) 2CG11 cells transiently transfected with HA-tagged NM full-length or NM mutant constructs. NM-HA was detected using MAb anti-HA (red). Some NM-GFP aggregates also stained with Hoechst, most likely due to nucleic acids incorporated in the aggregates. Mutant NM-HA Δ 39-123 hardly ever coalesced with NM-GFP aggregates. Scale bars, 5 μm. (D) Shown is the percentage of NM-GFP^agg^ cells with NM-HA aggregates compared to NM-GFP^agg^ cells expressing NM-HA (soluble or aggregated). Bars represent mean values ± SEM (four independent transfection experiments; *n* = 4). At least 630 cells were analyzed per cell population. Statistical analysis was done using the Cochran-Mantel-Haenszel test (*, *P* ≤ 0.05; ****, *P* ≤ 0.0001; ns, not significant). (E) Percentage of NM-GFP/NM-HA aggregate-bearing cells (D) that show colocalization of NM-HA and NM-GFP aggregates. Bars represent mean values ± SEM. Statistical analysis was done using the Cochran-Mantel-Haenszel test (****, *P* ≤ 0.0001; ns, not significant).

To determine if preexisting endogenous NM-GFP prions could sequester NM-HA mutants, N2a NM-GFP^agg^ cells were transiently transfected with empty vector or constructs coding for NM-HA wild type or NM-HA mutants (Fig. 5A). Note that NM-GFP^agg^ cells produce both long fibril-like and punctate NM-GFP aggregates (Fig. 5B). When transfected with constructs coding for NM-HA derivatives, HA-tagged NM aggregates were found in all cell populations (Fig. 5C). At least 36% of NM-GFP^agg^-bearing cells in all NM-HA derivative-expressing cells contained NM-HA aggregates independent of the mutant (Fig. 5D). Lowest NM-HA aggregate induction was found when parts of the OPR and the CTN (Δ 39-123 and Δ 75-123) were deleted. In cells exhibiting both NM-GFP and NM-HA derivative aggregates, NM-HA Δ 39-123 hardly ever decorated NM-GFP aggregates (Fig. 5C and E). Deletion of either the last 2.5 OPR or the CTN (Δ 75-97, Δ 75-123, or Δ 98-123) drastically decreased coaggregation with full-length NM. Instead, aggregated mutants often appeared as individual puncta (Fig. 5C and E). The presence of independent NM-HA mutant aggregates in cells producing NM-GFP prions suggests that prions lacking the last 2.5 repeats and the CTN or the CTN alone are structurally less compatible with full-length NM prions. Collectively, these data suggest that parts of the OPR and the CTN mediate intermolecular contacts between soluble NM with preexisting exogenous or endogenous NM seeds.

### Vertical transmission of NM aggregates to progeny.

**FIG 6:**
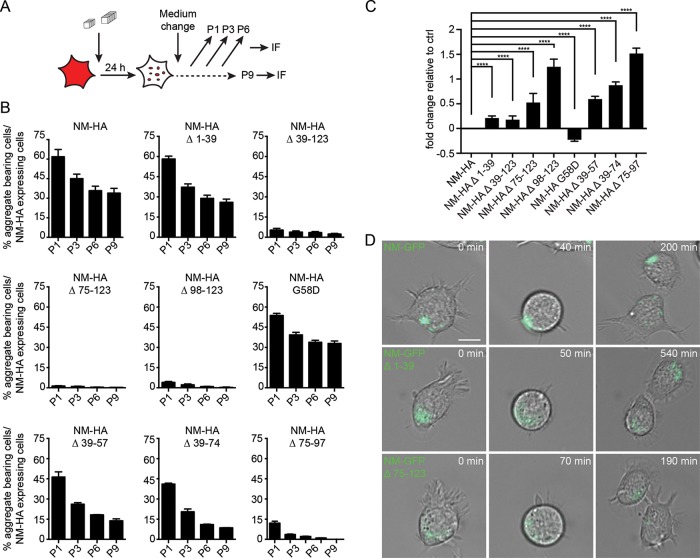
Maintenance of NM-HA aggregates over multiple cell divisions. (A) Experimental setup to study aggregate maintenance over several cell passages. In a given population, the number of cells stably expressing the transgene was determined for each passage. These cells were then analyzed for NM-HA aggregates. (B) Percentage of cells expressing wild-type or mutant NM-HA that harbor aggregates over multiple cell divisions. Cell populations were exposed to 10 μM untagged recombinant NM fibrils (monomer concentration) and subsequently passaged every 2 to 3 days. Cells with aggregates were determined by automated confocal microscopy analysis (*n* = 3 biological replicates). Bars represent mean values ± SEM. P, passage. At least 7,900 cells were analyzed per cell population per passage. Proliferation doubling time for N2a NM-HA cells is approximately 13 h (data not shown). This corresponds to approximately 51 doublings within 13 passages. (C) Fold change of the probability for NM-HA mutant cell populations to lose aggregate-harboring cells over time compared to cells expressing full-length NM-HA. >0 denotes lower mitotic stability than NM-HA, and <0 denotes higher mitotic stability than NM-HA. Bars represent mean values, and error bars represent the upper limit for the confidence interval (*n* = 3). The Cox proportional hazard model was used for statistics (cell count, >12,500; ****, *P* ≤ 0.0001). (D) Time-lapse analysis of N2a cell lines stably expressing NM-GFP full-length, Δ 1-39, or Δ 75-123. Cells were exposed to 10 μM untagged recombinant NM fibrils for 24 h (monomer concentration). Two passages postinduction, cells were subjected to live cell imaging for a total of 15 h. Scale bar, 10 μm.

A hallmark of mammalian and yeast prions is their stable maintenance in dividing cell populations (21, 53). The persistence of aggregates over multiple cell divisions is taken as a proxy for aggregate multiplication (22, 53). We compared the number of cells bearing NM-HA aggregates to the number of cells expressing NM-HA over continuous passages after fibril exposure (Fig. 6A and B). Independent of the NM-HA construct, the number of aggregate-bearing cells decreased over time, but loss appeared to slow down between passages six and nine for full-length NM-HA, mutant G58D, and NM-HA aggregates lacking the QNR (Fig. 6B). The initial presence of unstable prion variants has been observed in yeast (31, 54). Less than 13% of cells with aggregates were observed for mutants lacking the last 2.5 repeats and/or the CTN (Δ 39-123, Δ 75-97, Δ 75-123, and Δ 98-132). As spontaneous aggregate induction was less than 0.3% except for the NM-HA mutant without the QNR (Fig. 3A to D), the persistence of aggregates suggests that at least a subpopulation of induced aggregates was able to propagate. We used the Cox proportional hazard model to assess the fold change in the loss of aggregate-bearing cells over multiple cell divisions. A higher fold change in cells with mutant NM aggregates compared to cells with wild-type NM aggregates (set to zero) was taken as a measure for lower mitotic stability (Fig. 6C). Generally, deletions in NM decreased the mitotic stability of aggregates. NM-HA Δ 75-97 and Δ 98-123 aggregates were lost most frequently, suggesting that the last 2.5 repeats and the CTN are most important for stable inheritance. NM-HA G58D showed increased mitotic stability compared to that of full-length NM-HA (Fig. 6C). This mutation has been shown to modulate prion activity dependent on prion variant as well as genetic background of the yeast host by affecting either seed fragmentation or transmission to progeny (26, 49, 55). To monitor the fate of induced NM aggregates upon cell division, N2a cell lines were generated that stably express full-length NM or NM mutant Δ 1-39 or Δ 75-123 fused to GFP (Fig. 6D). Aggregation was induced by exposure to untagged recombinant NM fibrils. Distribution of NM-GFP aggregates to both daughter cells also confirmed that mutant NM aggregates that lack either the QNR or the last 2.5 repeats and the CTN are bidirectionally transmitted to daughter cells. We conclude that deletion of the last 2.5 repeats and the CTN had strongest effects on mitotic prion stability.

### NM mutants are infectious.

**FIG 7:**
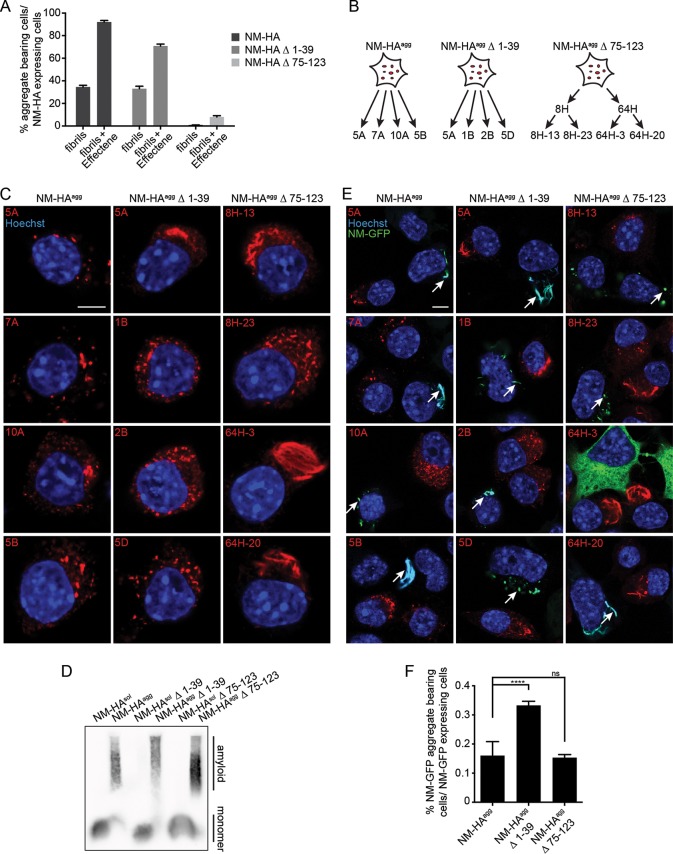
Induction of the prion state in bystander cells. (A) Untagged recombinant NM fibrils (10 μM monomer concentration) were either added directly or mixed with Effectene transfection reagent before addition to full-length and mutant NM-HA cell populations. Induction efficiency was determined at passage three postexposure. At least 400 cells per treatment were examined. Bars represent mean values ± SEM. (B) Cloning strategy to isolate N2a clones with stable NM aggregates. Cell populations were exposed to 10 μM untagged recombinant NM fibrils (monomer concentration) with Effectene for 24 h. Cell clones with NM-HA aggregates were subsequently isolated by limiting dilution cloning. For mutant NM-HA Δ 75-123, subcloning was required to isolate four subclones with stable aggregate propagation. (C) Confocal microscopy analysis of cell clones isolated by limiting dilution cloning. For NM-HA wild type and both NM-HA mutants, four clones/subclones were analyzed. NM was detected using MAb anti-HA (red). Nuclei were stained with Hoechst (blue). Scale bar, 5 μm. (D) SDD-AGE assay of cell lysates from N2a NM-HA^agg^ clone 7A, Δ 1-39 clone 5D, Δ 75-123 subclone 8H-23, and the corresponding soluble bulk cell lines. NM-HA was detected using MAb anti-HA. (E) Clones with NM-HA aggregates were cocultured with recipient cell line N2a NM-GFP^sol^ for 42 h. Cells were analyzed for recipient cells with NM-GFP aggregates (arrows). NM-HA was detected using MAb anti-HA (red). Nuclei were stained with Hoechst (blue). GFP is shown in green. Note that large NM-GFP aggregates also stained brightly with Hoechst dye. Scale bar, 5 μm. (F) Percentage of recipient cells harboring NM-GFP aggregates after 42 h of coculture with donor clones. Bars represent mean values of the combined data from the four NM-HA clones/subclones ± SEM (*n* = 4). Cell count, >79,000; ****, *P* ≤ 0.0001; ns, not significant. Statistical analysis was done using the Cochran-Mantel-Haenszel test.

Contrary to known behavior in S. cerevisiae, NM prions in mammalian cells infect naive bystander cells (24). To explore if NM deletion mutants exhibit infectious properties, we first generated donor populations with a higher number of aggregate-bearing cells by transfecting untagged recombinant NM fibrils into N2a NM-HA^sol^, Δ 1-39^sol^, and Δ 75-123^sol^ cell populations using Effectene (Fig. 7A). Cloning was subsequently performed (Fig. 7B) to isolate cell populations with high prion replication ability (56). A total of 24 N2a NM-HA clones was isolated, 14 of which produced aggregates in 80 to 100% of total cells. For NM-HA Δ 1-39, 16 out of 20 clones stably propagated aggregates. For NM-HA Δ 75-123, the initial round of cloning generated only 2 out of 144 clones with aggregates in only 40 to 70% of cells, suggesting mitotic instability. Subcloning generated 86 clones, with 21 clones harboring visible aggregates in at least 50% of the cells. Four clones/subclones were chosen for the NM-HA wild type and the two NM-HA mutants that exhibited a high percentage (≥80%) of cells harboring aggregates (Fig. 7B). Cell clones displayed punctate aggregates that were evenly distributed throughout the cytosol or clustered in certain areas, and occasionally large, fibrillar aggregates were observed (Fig. 7C). SDD-AGE analysis (23) confirmed high-molecular-weight NM aggregates (Fig. 7D), demonstrating that NM deletion mutants also were able to form amyloid fibrils (57).

All clones, except one subclone producing large aggregates (NM-HA Δ 75-123; clone 64H-3), induced aggregation of NM-GFP in recipient cells upon direct coculture (Fig. 7E). Consistent with our previous findings, intercellular NM prion induction was a rare event (24). Generally, intercellular induction appeared to be higher when donor cells produced small, punctate aggregates. We were unable to detect donor NM-HA aggregates in the recipient cells, as transmitted, seeding-competent NM polymers are usually too small to be visualized by confocal microscopy (25). A significantly increased induction rate was found for aggregates lacking the QNR compared to full-length NM; however, clonal differences cannot be excluded (Fig. 7F) (24). In summary, neither the QNR nor the last 2.5 repeats and the CTN are essential for intercellular aggregate transmission.

### Efficiently propagating NM prions contain a carboxy-terminal protease-resistant core.

**FIG 8:**
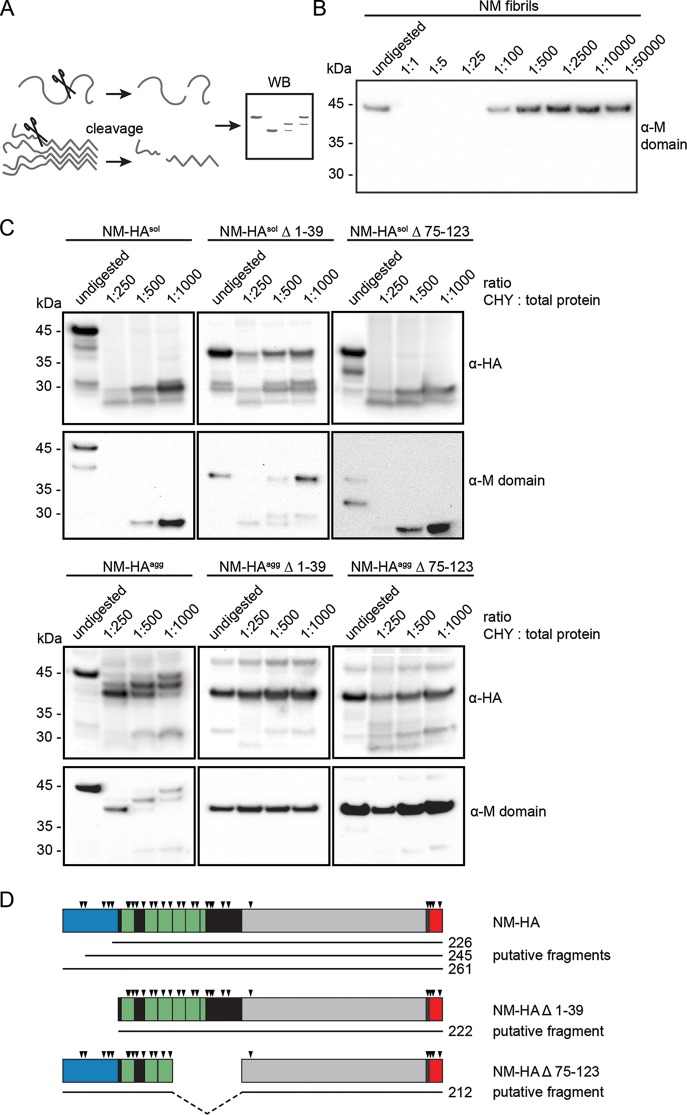
Partial protease resistance of wild-type and mutant NM-HA. (A) Experimental setup to define the protected aggregate core of wild-type and mutant NM-HA by enzymatic proteolysis. (B) Limited proteolysis of *in vitro*-fibrillized untagged recombinant NM. Fibrils were treated with chymotrypsin at the indicated protease/total protein ratio for 1 h on ice or were left untreated. Proteins were analyzed by Western blotting. NM was detected by 4A5 anti-M domain antibody (epitope, aa 229 to 247). (C) Limited proteolysis of N2a NM-HA^sol/agg^ wild type, Δ 1-39, and Δ 75-123 cells. Lysates of N2a NM-HA^agg^ wild-type clone 7A, Δ 1-39 clone 5D, Δ 75-123 subclone 8H-23, and the corresponding bulk cell lines expressing soluble NM-HA were subjected to chymotrypsin at the indicated protease/total protein ratio or left untreated. Proteins were analyzed by SDS-PAGE and Western blotting. NM was detected by anti-HA antibody or 4A5 anti-M domain antibody. Differences in exposure times are indicated by separate boxes. (D) Putative fragments of full-length and mutant NM after limited proteolysis with chymotrypsin. Arrowheads indicate putative cleavage sites of chymotrypsin. Blue, QNR; green or black, OPR and CTN; gray, M domain; red, HA tag. Numbers refer to amino acid residues.

The amyloid core of Sup35 prions propagating in S. cerevisiae has been experimentally mapped to the QNR (58). We used limited proteolysis (Fig. 8A) to characterize differences in prion cores of NM-HA wild type and Δ 1-39 and Δ 75-123 mutants (59). Chymotrypsin preferentially cleaves at the carboxyl side of amide bonds of tyrosine, tryptophan, and phenylalanine. These residues are abundant in the N domain, but except for one residue (aa 129), they are not present in the M domain. Three tyrosine residues are also part of the HA antibody epitope tag. As expected, chymotrypsin cleavage sites became inaccessible to partial proteolysis in the tightly packed untagged recombinant NM fibril (Fig. 8B). When cell lysates of N2a cells with soluble or aggregated (Fig. 8C) full-length or mutant NM-HA derivatives were incubated with chymotrypsin, polymers exhibited higher resistance to proteolysis. Proteolysis of full-length NM-HA^agg^ resulted in a sequential decrease in protein size, suggesting that amino-terminally located cleavage sites were accessible to proteases (Fig. 8C). Increasing chymotrypsin concentrations resulted in the appearance of a wild-type NM-HA^agg^ band at approximately 38 kDa, a size approximately similar to that of the NM deletion mutants. Both NM-HA^agg^ mutants Δ 1-39 and Δ 75-123 sustained proteolysis, generating approximately similarly sized fragments (Fig. 8C). Very weak low-molecular-weight bands of NM-HA Δ 75-123 most likely represent proteolytic fragments of residual soluble protein (compare to Fig. 8C, top panel). These findings argue that the entire carboxy termini of wild-type and mutant NM, including the HA tag, were protected (Fig. 8D). The involvement of the M domain in the amyloid core has recently been reported for some Sup35 prions in S. cerevisiae (60, 61). The fact that we observed similarly sized fragments for the chymotrypsin-treated NM-HA^agg^ wild type and the undigested NM-HA^agg^ deletion mutants that still harbor the carboxy-terminal HA tag strongly suggests that (i) the QNR in full-length NM-HA^agg^ does not engage in the tightly packed core, (ii) aggregated NM-HA full-length and mutant NM-HA Δ 1-39 likely share a similar core, and (iii) NM-HA^agg^ Δ 75-123 has a different amyloid core that also includes the QNR.

## DISCUSSION

**FIG 9:**
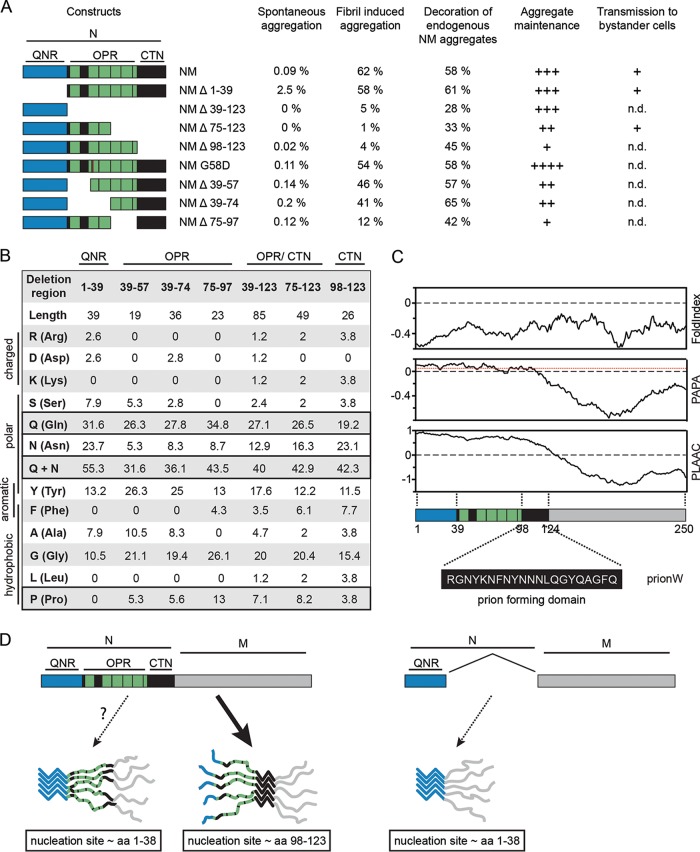
Carboxy-terminal OPR and the CTN are involved in nucleation, template-assisted aggregation, and prion maintenance. (A) Summary of results. Spontaneous aggregation, template-assisted aggregation, maintenance, and transmission to bystander cells by bulk cell populations or cell clones stably expressing the respective NM-HA proteins. Coaggregation with endogenous NM-GFP aggregates was determined following transient overexpression of wild-type and mutant NM-HA in N2a NM-GFP^agg^ cells. Shown is the percentage of cells showing NM-GFP/NM-HA aggregate colocalization per NM-GFP/NM-HA aggregate-bearing cell. Aggregate maintenance was assessed as a proxy for seed multiplication: ++++, slightly increased mitotic stability compared to NM-HA^agg^ control cells (fold change, >−0.5 and <0); +++, only slightly altered mitotic stability compared to NM-HA^agg^ control (fold change, >0 and <0.5); ++, lower mitotic stability compared to control (fold change, >0.5 and <1); +, lowest mitotic stability (fold change, >1 and <2). For transmission to bystander cells, a plus sign indicates that at least 1 in 1,000 recipient cells contained NM-GFP^agg^. n.d., not done. (B) Amino acid composition of different subdomains of the N domain. The position of the region within NM and its length are indicated at the top. The abundances of specific residues within the region are given as percentages. (C) Prediction of disorder (FoldIndex [86]) and prion domains according to PAPA (16), PLAAC (15), or pWaltz/PrionW (80). Images were designed using PLAAC (http://plaac.wi.mit.edu/). The region identified as the prion nucleating domain by PrionW is shown at the bottom. This region falls within the CTN (aa 98 to 123). (D) Putative model of NM prion nucleation and seeding in N2a cells. NM contains two putative nucleation sites. Within the mammalian cytosol, the carboxy-terminal site comprising aa residues 98 to 123 preferentially drives nucleation and template-assisted seeding (left). The amino-terminal nucleation site (residues 1 to 38) likely mediates self-recognition and assembly when the carboxy-terminal site is deleted (right).

Q/N-rich PrLD reminiscent of prion domains of lower eukaryotes are abundant in the proteomes of higher eukaryotes (17, 18). Experimental evidence is lacking that their putative PrLDs can turn the protein into a bona fide prion. To understand in more detail the sequence features in Q/N-rich domains that can drive prion formation in mammalian cells, we made use of the prototype yeast prion domain Sup35 NM that also amplifies as a prion in the mammalian cytosol (23–25). Strikingly, subdomains that preferentially drive the sequential steps of prion biogenesis differ between S. cerevisiae and our murine cell line (Fig. 9A). In S. cerevisiae, the QNR mediates *de novo* prion induction (11, 32). This region is crucially required for propagation of all so-far tested wild-type Sup35 prion variants in S. cerevisiae, and its deletion abrogates prion activity (11, 32, 37, 47, 48, 62). In N2a cells, the QNR was not required for prion induction. Instead, *de novo* NM aggregate induction, exogenous and endogenous NM seed-induced prion formation, and maintenance were preferentially driven by the last 2.5 repeats of the OPR and the CTN. Depending on the prion variant and/or genetic background of the host, the minimal region required for stable prion maintenance in S. cerevisiae varies but consistently includes the amino-terminal OPR (32, 37, 42, 45–48) proposed to enhance fibril fragmentation by disaggregase Hsp104 (32, 50, 51, 63, 64). As Hsp104 has no homologue in the mammalian cytosol (65), NM seed multiplication in N2a cells must proceed by other means. Potentially, the metazoan Hsp70 machinery in conjunction with Hsp110 (66) or autophagy-related processes contribute to prion partitioning (67). Alternatively, NM prions might also form through a fibril-catalyzed secondary nucleation of oligomers, a process reported for Aβ-42 seed proliferation (68).

The amino-terminal QNR (approximately aa 9 to 39) and the CTN (approximately aa 90 to 120) have been identified recently as short recognition elements that mediate intermolecular contacts during *de novo* amyloid assembly and in mature fibrils generated *in vitro* (69, 70). Accumulating evidence suggests that Sup35 prion variants are composed of distinct parallel and in-register superpleated β-structures that differ in the extent of their β-sheets (58, 60–62). Recombinant full-length NM fibril preparations contain mixtures of conformers (35, 71) with QNR-containing amyloid cores (58, 60, 72) that induce prion variants with similar core structures in S. cerevisiae (37, 38). However, amyloid fibrils generated from a synthetic NM peptide (aa 98 to 118) can induce a Sup35 prion phenotype when transformed into S. cerevisiae. It is unclear if the induced prion variant exhibits a tightly packed core extending to the QNR as other variants in yeast do (73). The emergence of the new wild-type NM prion variant in N2a cells that lacks the QNR in its tightly packed core could be due to the selective amplification of few NM conformers with a carboxy-terminal amyloid core already present in the recombinant fibril mixture. Alternatively, initially induced NM prions with amino-terminal amyloid cores could interconvert into conformers with greater fitness in mammalian cells (74, 75). The reason for increased fitness of NM prions with tightly packed cores lacking the QNR is unknown but could relate to the cellular machinery involved in fibril assembly and/or maintenance or inhibitory factors. NM prions propagating in N2a cells exhibit reduced infectivity in yeast, potentially because this prion variant is not adapted to the yeast disaggregating machinery (23). In line with this, a recent study showed that yeast Hsp104 inefficiently fragments NM prions whose amyloid core extends to the carboxy-terminal N region (76). So far, however, molecular mechanisms or cofactors required for prion biogenesis in mammals are ill defined (77).

Bioinformatic predictions of proteins with prion-like behavior have been notoriously difficult (10, 12–14, 78). Importantly, prion prediction algorithms have been developed and trained on Q/N-rich yeast prion domains active in S. cerevisiae. While even for lower eukaryotes developed prion predictors suffer from significant limitations (10), predictions can be even more challenging when it comes to predicting the aggregation propensity of human PrLD-containing proteins in more complex organisms (20). PLAAC and PAPA algorithms support the compositional model that predicts that weak intermolecular interactions over a long Q/N-rich disordered domain confer prion propensity (15, 16). pWaltz (webserver PrionW) postulates that a short sequence in the disordered Q/N-rich context acts as a soft amyloid core that mediates prion assembly (79, 80). Importantly, the putative amyloid core element identified by pWaltz/PrionW (aa 98 to 118) (Fig. 9B and C) is basically identical to the CTN that, together with the last 2.5 repeats, is involved in spontaneous and fibril-assisted prion induction in our N2a cell model. The CTN could therefore act as the nucleation site for initial assembly, while the adjacent repeats stabilize intermolecular contacts within the aggregate (Fig. 9D) (45). Collectively, our results are in line with the hypothesis that short amyloidogenic stretches embedded in Q/N-rich disordered domains drive prion assembly.

## MATERIALS AND METHODS

### Cell culture and reagents.

N2a cells were grown in Dulbecco's modified Eagle's medium (DMEM) (Gibco) supplemented with 10% fetal bovine serum (FBS) (PAA). HEK 293T/17 cells were grown in Iscove's modified Dulbecco's medium (Sigma) supplemented with 1% glutamine, 10% FCS, and 3.024 g/ml NaHCO_3_. Chemicals were purchased from Sigma or Carl Roth, unless stated otherwise. Monoclonal antibody (MAb) rat anti-HA clone 3F10 (1:1,000) (Roche Diagnostics), MAb 4A5 rat anti-NM (M domain) (1:10) (81), MAb mouse anti-Myc epitope (Miltenyi Biotec), MAb antiactin clone C4 (1:5,000) (MP Biomedicals), MAb mouse anti-HA (1:200) (Santa Cruz), MAb mouse anti-HA–Alexa Fluor 647 (1:500) (Biozol), or polyclonal antibody (pAb) rabbit anti-Myc tag (Abcam) was used. Horseradish peroxidase-conjugated antibodies were from Dianova, and fluorescently labeled antibodies were from Life Technologies. Cytoplasm was stained with HCS CellMask (Life Technologies). Effectene (Qiagen) or Lipofectamine 2000 (Thermo Scientific) was used for transfection.

### Generation of NM-HA- and NM-GFP-expressing cell lines.

Constructs for N, M, NM Δ 138-250, and NM Δ 39-123 were ordered from Thermo Scientific or DNA 2.0. All other NM constructs were generated by PCRs with mutation-specific primers using the previously described NM-HA construct (33) as the template. Carboxy-terminally Myc- or HA-tagged constructs were cloned into the mammalian expression vector pJ602 and the lentiviral vector pRRL.sin.PPT.hCMV.Wpre (82). Gateway technology was used to generate lentiviral plasmids coding for GFP-tagged full-length NM and NM mutants (24). Viral particles were produced as described previously and used for transduction of N2a cells (24).

### Sedimentation assay.

Cells were incubated with 10 μm *in vitro*-fibrilized NM (monomer concentration) for 24 h or left untreated. Cleared cell lysates (10 mM Tris-HCl, pH 7.5, 100 mM NaCl, 10 mM EDTA, 0.5% Triton X-100, 0.5% sodium deoxycholate, protease inhibitors) were centrifuged at 20,000 × *g* for 20 min at 4°C (the pellet was the insoluble fraction). Supernatant fractions were precipitated with 4× methanol at −20°C, and protein was pelleted at 2,120 × *g* for 25 min at 4°C (soluble fraction). The insoluble fraction and 1/6 of the soluble fraction dissolved in TNE buffer (50 mM Tris-HCl [pH 7.4], 100 mM NaCl, 0.1 mM EDTA) were loaded. Protein was detected using Pierce ECL (Life Technologies) or ECL Prime (GE Healthcare).

### SDD-AGE.

The semidenaturing detergent-agarose gel electrophoresis (SDD-AGE) assay was performed as described previously (83). Cells were lysed (50 mM Tris-HCl, pH 7.5, 150 mM NaCl, 1% NP-40, complete protease inhibitor) for 30 min on ice. Cleared supernatant was mixed with 4× sample buffer (2× TAE, 20% glycerol, 8% SDS, bromphenol blue), incubated at room temperature for 5 min, and separated on a 1.5% agarose gel supplemented with 0.1% SDS. Proteins were transferred onto a nitrocellulose membrane using capillary transfer.

### Immunofluorescence staining and confocal microscopy analysis of N2a cells.

Cells were fixed with 4% paraformaldehyde and permeabilized for 10 min (0.5% Triton X-100). Antibodies were added in 2% blocking solution overnight at 4°C. Nuclei were visualized with Hoechst 33342 (1:10,000). Confocal microscopy was performed using an LSM 700 or LSM 710 (Zeiss, Jena, Germany). Cells on 96-well plates were stained with HCS CellMask blue stain (1:5,000), imaged with an automatic Cell Voyager 6000 confocal microscope (Yokogawa), and analyzed with Cell Profiler cell image analysis software. Time-lapse images of N2a NM-GFP cells were taken two passages after fibril exposure over a period of 15 h with an ApoTome (Zeiss) at 37°C and 5% CO_2_.

### Aggregate induction by recombinant NM fibrils.

Untagged recombinant NM protein was prepared as described previously (24). To generate fibrils, NM was rotated head over tail for 24 h at 4°C at a concentration of 10 μM to 100 μM (monomer concentration). NM fibrils were sonicated for 3 min and added to cells (end concentration, 10 μM; monomer concentration). After 24 h the medium was exchanged.

### Generation of N2a full-length NM-HA^agg^ and mutant cell clones and coculture.

To enhance the fibril induction rate, untagged recombinant fibrils were mixed with the appropriate amount of Effectene transfection reagents (Qiagen) used for transfections in 24-well plates prior to addition to N2a cells. Cells were cultured for three passages, and the percentage of aggregate-bearing cells was determined. Aggregate-bearing cell clones were isolated. For NM-HA^agg^ Δ 75-123, a second cloning step was necessary, as the first round yielded clones with a lack of mitotic stability. N2a NM-HA^agg^ donor clones were cocultured with N2a NM-GFP^sol^ cells (ratio, 1.5:1). After 42 h, cells were fixed and stained as described above. Confocal images were taken with an LSM700 (Zeiss) or Cell Voyager 6000.

### EM analysis of fibril morphology.

Ten μM untagged recombinant NM fibrils (monomer concentration) was transferred to glow-discharged Formvar/carbon-coated electron microscopy (EM) grids (Plano GmbH). Samples were contrasted in 2% uranyl acetate and examined using a JEM-2200FS transmission electron microscope (JEOL) at 200 kV.

### Image data analysis.

Confocal images were obtained using a Cell Voyager CV6000 with ×40 magnification. For NM aggregate colocalization, the LSM 700 with ×63 magnification was used. Images were analyzed with Cell Profiler. Nuclei and the associated cytoplasm were identified based on morphology parameters and intensities of Hoechst 33342 and HCS CellMask. NM-expressing cells were identified using intensity levels of immunofluorescently labeled NM. Aggregates were classified using the Ilastik segmentation tool kit. The numbers of wild-type or mutant NM-expressing cells and aggregate-bearing cells were determined.

### Statistical analysis.

Individual experiments were performed at least three times, and for statistically analyzed data at least 550 cells per cell population were imaged. All data displayed in bar graphs are depicted as the means. Error bars represent the standard errors of means (SEM) or the upper limit of the confidence interval. *P* values of ≤0.05 were considered significant.
